# Sociodemographic, Clinical, and Variation Outcomes for Breast Cancer and Breast Cancer-Related Mutations in a Ten-Year Cohort From Neiva, Huila, Colombia

**DOI:** 10.7759/cureus.32257

**Published:** 2022-12-06

**Authors:** Justo Olaya, Juan Sanjuan, Diana Torres-Lopez, Laura Olaya, Miguel Gutierrez-Vargas, German Olaya, Juan Diego Olaya

**Affiliations:** 1 Surgery - Mastology, Universidad Surcolombiana, Neiva, COL; 2 Surgery - Mastology, Hospital Universitario Hernando Moncaleano Perdomo, Neiva, COL; 3 Surgery - Mastology, Unidad Oncologica Surcolombiana, Neiva, COL; 4 Clinical Research, Unidad Oncologica Surcolombiana, Neiva, COL; 5 Clinical Research, Epidemiologos SAS, Cali, COL; 6 Intensive Care, Universidad del Valle, Cali, COL; 7 Intesive Care Unit, Clinica Imbanaco - Grupo Quirón Salud, Cali, COL; 8 Clinical Research, Cirugia y Trauma (CYTRA) - Universidad Surcolombiana, Neiva, COL; 9 Genetics, Universidad Javeriana, Bogotá, COL; 10 General Medicine, Unidad Oncologica Surcolombiana, Neiva, COL; 11 General Medicine, Fundación Universitaria De Ciencias De La Salud, Bogotá, COL

**Keywords:** brca1/brca2, hereditary cancer, genetics, molecular biology, brca founder mutation, breast cancer gene, genetic breast cancer

## Abstract

Background

Some breast cancer cases are related to inherited mutations, and this is the reason why early mutation screening is emerging as an area of focus for cost-effective care. However, breast cancer-related mutations vary according to race, ethnicity, geographic origin, and healthcare access. Surveillance for familial breast cancer is not performed routinely in Colombia. Our main aim in this study was to describe a cohort of breast cancer patients, carrying founder breast cancer gene (BRCA) mutations, which were followed up for up to 10 years (2010-2019) in Neiva, Colombia.

Methods

We performed a retrospective description from an outpatient care center in Huila, Colombia, a region with high breast cancer rates. This study included patients with both a breast cancer diagnosis and an incident genetic mutation for breast cancer (detected during a breast cancer consultation). We captured information from patient medical records. Descriptive analyses were performed.

Results

A total of 105 patients met the study’s inclusion criteria and were included patients with the BRCA1 mutation and three with BRCA2 mutations. They had a median age of 45 years (IQR, 36 to 51 years). Relatives with a breast cancer history were found in 74 carriers (70.5%). Most patients had a report of Breast Imaging-Reporting and Data System (BIRADS) ≥ 4. A TNM (tumor, node, metastasis) changed reclassification was observed in anatomical vs. prognostic classification. Median follow-up was of 74 months (IQR, 44 to 130), overall observed mortality was 22.9%, and specific mortality was 19.1%.

Conclusion

Women with breast cancer who carry a mutation related to breast cancer are usually younger than age 50 at diagnosis. Developing strategies and specific policies for this population is needed, and a prevalent BRCA1 c.3331_3334delCAAG mutation could be used as a cost-effective first approach. Among these patients, a risk-increased reclassification was observed.

## Introduction

In Colombia, 15,509 new breast cancer cases were diagnosed in 2020 [1]. The average age for breast cancer diagnosis is 59.5 years [2]. Colombian women under age 40 have a higher risk for breast cancer when compared to women in the United States and Canada [1]. Colombian breast cancer patients younger than age 40 tend to have a higher proliferation index, a lower hormone reception expression, and genetic mutations, which are important factors concerning breast cancer [3,4].

Approximately 10% of all breast cancer cases are related to genetic factors [5,6]. These loss-of-function germline mutations in tumor suppressor genes vary according to cancer susceptibility. These mutations can be classified as high-penetrance mutations (BRCA1, BRCA2, TP53, PTEN, STK11, and CDH1) and moderate penetrance variants (CHK2, ATM, RAD51C, BRIP1, and PALB2) [6]. Particularly regarding BRCA mutations, most patients are younger than 35 years old and have worse clinical manifestations, tumor features, and prognosis [7,8]. However, the risk gap between them is closing due to advancements in screening and treatments. Specific mutation factors in BRCA carriers, such as positive regional lymph nodes, increased primary tumor size, age, and negative receptor status, cause survival rates to vary [7]. Most of these factors correlated to a significant impact on later disease stages [8]. Given this consideration, early mutation detection is emerging as an area of focus for cost-effective care [9,10]. However, a recently published study of clinical genetic testing and outcomes among patients with breast and ovarian cancer in the US showed that nearly 25% of those with breast cancer had genetic test results of note with variation according to race or ethnicity and insurance [11]. The study included insured and uninsured out-of-pocket charges and elucidated testing disparities, limiting the interpretation of breast cancer patient's genetic results. Surveillance for familial breast cancer does not exist in Huila, Colombia. Patients with a very strong family history of breast or ovarian cancers are not screened routinely, which leads to preventable delays in diagnosis. Our main aim is to describe sociodemographic factors, clinical factors, and outcomes in patients with breast cancer and breast cancer-related mutations in Neiva, Huila, Colombia, over ten years from 2010 to 2019.

## Materials and methods

Patient population

We performed a retrospective observation and analytical cohort study at our specialized oncology outpatient care center in Huila, Colombia, a region with high reported breast cancer rates. Huila is a city of 1.1 million people and our specialized medical center diagnoses and treats ~70% of all breast cancer patients in the region. Patients included in the study were referred to our center and live in Colombia's southern regions. This study included patients with both a breast cancer diagnosis and an incident genetic mutation for breast cancer (detected during breast cancer consultation). We captured clinical and sociodemographic information from patient medical records, such as age at diagnosis and insurance, which delimited our population. Also, we collected participant clinical status at the diagnostic time, tumor features, the diagnostic process (imaging), management, and outcomes features.

Statistical analyses

Statistical analyses were performed using Stata, version 15 (StataCorp LLC, College Station, Texas, USA). A descriptive statistic was performed, using categorical variables' absolute (n) and relative (%) frequencies. Median (Med) and interquartile range (IQR) were employed for continuous variables. Survival analyses were assessed using the Kaplan-Meier test. The center's institutional review board approved this study.

## Results

Demographics

A total of 1,378 female patients were screened, and 105 met the inclusion criteria of having both a breast cancer diagnosis and breast cancer-related genetic mutation. The carriers' median age was 45 years (IQR, 36 to 51 years). Approximately half of the patients reported urban work activities and undergraduate education. Approximately 25% of the carriers had subsidized insurance (Table 1).

**Table 1:** Sociodemographic characteristics in patients with breast cancer and breast cancer-related mutation in Neiva, Huila, Colombia, from 2010 to 2019 ^a^Median IQR, Interquartile Range

	n	Percentage
Age (years)^a^	45	(36 - 51)
Ethnicity		
Indigenous	2	1.9%
Admixed?	103	98.1%
Population Characteristics		
Urban Worker	53	50.5%
Other	37	35.2%
Older people	9	8.6%
Householder	4	3.8%
Region		
Huila	98	93.3%
Caquetá	4	3.81%
Cauca	1	0.95%
Putumayo	1	0.95%
Insurance		
Contributory	63	60.0%
Subsidized	26	24.8%
Special	16	15.2%
Education		
Elementary	44	41.9%
High School	14	13.3%
Technical	12	11.4%
College	29	27.6%
Postgraduate	6	5.7%

Breast cancer risk factors

The reported median age of menarche was 13 years (IQR, 12 to 14 years old). A total of 97 patients (92.4%) reported at least one pregnancy, and the total pregnancies were two (IQR, 2 to 3 pregnancies) with a median age of first pregnancy of 21 years (IQR, 18 to 25 years). Breastfeeding was reported by 89 patients (84.8%). The use of oral contraceptives (n=35; 33.3%) and hormone therapy (n=2; 1.9%) was also identified. Lifestyle risk factors in this cohort were smoking (n=11; 10.5%), occasional alcohol consumption (n=100; 95.2%), and physical activity (n=7; 6.7%). We found 32 previous malignancies reported, consisting of previous breast cancer (n=20; 19.1%), ovarian cancer (n=8; 8.6%), pancreatic cancer (n=2; 1.9%), and one patient with endometrium cancer and another patient with colon cancer (n=1; 0.95%).

Related familiar breast cancer record

Relatives with a breast cancer history were found in 74 carriers (70.5%). One family member with breast cancer was reported in 36 patients (34.3%), two family members with breast cancer were reported in 29 patients (26.7%), and three family members with breast cancer were reported in eight patients (7.6%). One carrier had four relatives with a history of breast cancer. Commonly reported relatives were aunts, cousins, and mothers (Table 2).

**Table 2:** Family members with breast cancer in patients with breast cancer and breast cancer-related mutation in Neiva, Huila, Colombia from 2010 to 2019

	N	Percentage
Family members with breast cancer	74	70.5%
None	31	29.5%
1	36	34.3%
2	29	27.6%
3	8	7.6%
4	1	0.9%

Diagnostic assessment

Mammography was performed in 89 carriers (84.8%), most of them with a Breast Imaging-Reporting and Data System (BIRADS) ≥ 4 category findings. According to ultrasonographic assessment, a total of 94 patients (89.5%) were described with BIRADS category ≥ 4 (Table 3).

**Table 3:** Imaging assessment in patients during breast cancer assessment and breast cancer-related mutation in Neiva, Huila, Colombia, from 2010 to 2019 BIRADS, Breast Imaging-Reporting and Data System

	N	Percentage
Mammography (BIRADS category)		
0	10	11.2%
2	6	6.7%
3	16	18.0%
4	38	42.7%
5	11	12.4%
6	8	9.0%
Breast ultrasonography (BIRADS category)		
2	2	1.9%
3	8	7.7%
4	79	76.0%
5	13	12.5%
6	2	1.9%

Pathology and immunohistochemical markers

All patients' biopsy analyses found ductal carcinoma except one patient with lobular carcinoma. Positive estrogen receptors were reported in 32 patients (30.5%), positive progesterone receptors were reported in 24 patients (22.9%), and positive Her2neu was reported in seven patients (6.7%). Sixty-six patients had triple-negative markers (62.9%). Fifty-six patients had a positive ki-67 index (data were only available for this variable in 58 carriers).

Breast cancer genetic mutations

BRCA1 was the most frequent mutation, found in 102 carriers (97.1%). Three patients had BRCA2 mutations. Genetic sequences are listed in Table 4.

**Table 4:** Breast cancer identified mutation in patients with breast cancer in Neiva, Huila, Colombia, from 2010 to 2019 BRCA, Breast Cancer; FS, Frameshift Mutation; MS, Missense Mutation; NS, Nonsense Mutation; NCBI, National Center for Biotechnology Information; BIC, Bean Improvement Cooperative

Gene	Mutation Nomenclature					Total
	NCBI		N (%)	BIC designation	Type	
	HGVS nucleotide	HGVS protein				
BRCA1	c.3331_3334del	p.(Gln1111fs)	92(87)	3450DEL4	FS	92
	c.4523G>A	p.(Trp1508^a^)	4(4)		NS	4
	c.5123C>A	p.(Ala1708Glu)	2(2)		MS	2
	c.3764dupA	p.(Asn1255Lysfs)	2(2)		FS	2
	c.1674del	p.(Gly559fs)	2(2)		FS	2
BRCA2	c.4889C>G	p.(Ser1630Ter)	3(3)		NS	3

Clinical and prognostic staging

According to clinical tumor-node-metastasis (TNM) staging, locally advanced disease (TNM staging ≥ IIb and <IV) was found in 83 patients (79.0%); just one carrier had evidence of distant disease. Locally advanced disease (TNM staging ≥ IIb and <IV) was found in 85 patients (81.7%), and one carrier had evidence of distant disease. The TNM IIIb stage was the most common among study participants. TNM distributions are listed in Table 5.

**Table 5:** Anatomic and prognostic staging according to AJCC in patients with breast cancer and breast cancer-related mutation in Neiva, Huila, Colombia, from 2010 to 2019 AJCC, American Joint Committee on Cancer

	Anatomic Staging AJCC 7		Prognostic Staging AJCC 8	
	N	Percentage	N	Percentage
Clinical Stage				
IA	4	3.8%	3	2.9%
IB	0	0	6	5.8%
IIA	17	16.2%	9	8.7%
IIB	39	37.1%	18	17.3%
IIIA	33	31.4%	8	7.7%
IIIB	9	8.6%	40	38.5%
IIIC	2	1.9%	19	18.3%
IV	1	1.0%	1	1.0%

Management

Neoadjuvant chemotherapy was administered in 72 patients (68.6%), and surgery was performed in 30 patients (28.6%) as the first option of treatment. Overall, chemotherapy was administered to 97 patients (92.4%), and all but one patient underwent surgery. Surgical management was performed as follows: 34 patients (32.4%) underwent breast-conserving surgery, and 70 patients (66.7%) underwent a mastectomy. Sentinel lymph node biopsy was performed on 11 patients, three of whom received a micrometastasis pathological specimen report. Axillary lymph node surgery was performed in 98 patients (93.3%); 36 had positive lymph node pathological specimen reports. Breast risk reduction surgery was performed on 42 patients. Breast reconstruction surgery was immediate in 28 patients (26.%) and deferred in 19 patients (17.9%). The most frequent reconstruction technique was autologous plus implant reconstruction in 43 patients (91.5%). The chemotherapy regimen was as follows: an anthracycline-based regimen was administered to 67 patients (63.8%), combined with taxanes in 34 patients and cyclophosphamide in 32 patients. Eight patients received no chemotherapy.

Radiotherapy Management

External beam radiotherapy was administered to 72 patients (68.6%).

Follow-up

**Figure 1:**
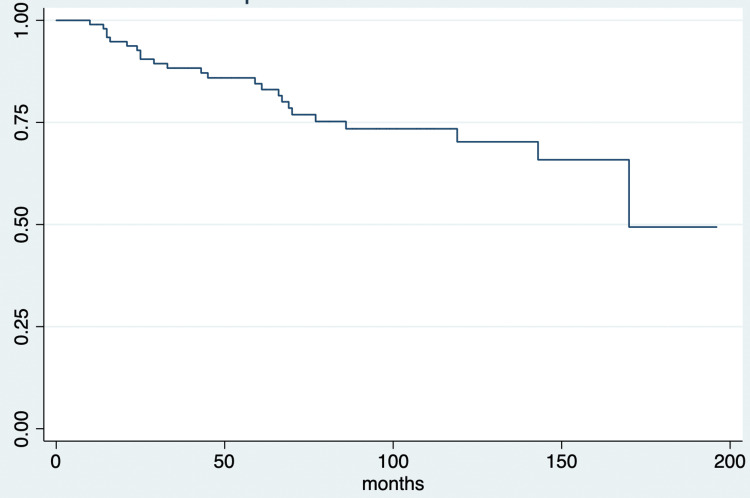
Survival estimates during the follow-up period in patients with breast cancer and breast cancer-related mutations in Neiva, Huila, Colombia, from 2010 to 2019

**Figure 2:**
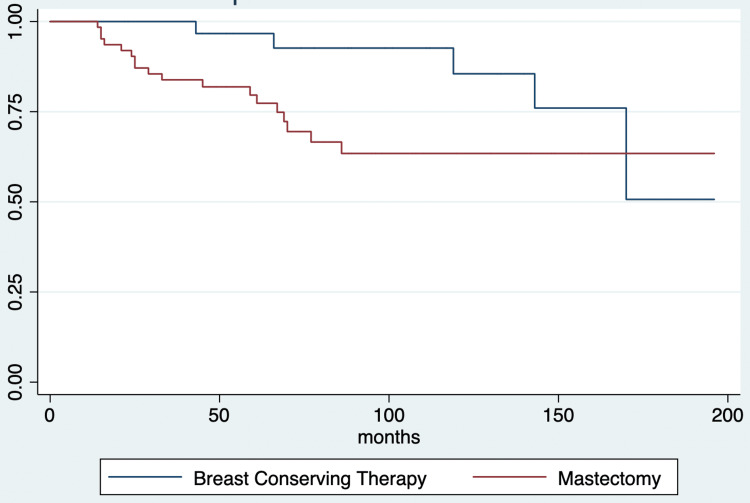
Survival estimates during the follow-up period according to the surgery management strategy in patients with breast cancer and breast cancer-related mutations in Neiva, Huila, Colombia, from 2010 to 2019

After a median follow-up of 74 months (IQR, 44 to 130 months), overall observed mortality was 22.9% (24 patients) and specific mortality was 19.1% (20 patients). The median time to death was 46 months (IQR, 14 to 69 months). By the end of the follow-up, two patients had active breast cancer disease. Twenty-six patients had other malignancy diagnoses (Figure 1, Figure 2).

## Discussion

BRCA-positive cancer has a differential risk and clinical features associated with poor prognoses such as sporadic cancer [7,8]. Results from various studies of patient characteristics and outcomes highlight that knowledge of the population's variability allows for a better understanding of this disease and improved strategies for its assessment [11]. Identifying relevant characteristics in a large population (especially in a high-risk region) is important for assessing cost-effectiveness strategies such as screening methods and early mutation staging [9-11]. Breast cancer data in Colombia are based on institutional experiences, limiting the understanding of breast cancer and breast cancer-related mutations at the population level [12-14]. Our institution is a referral center for oncology patient management in Southwestern Colombia. Our goal was to understand and describe breast cancer and its related mutation in a large population. Our results help assess the impact of strategies such as screening methods and mutation detection.

We performed a retrospective data analysis of patients with breast cancer and breast cancer-related mutations in Neiva, Huila, Colombia, over 10 years. Our report included population-risk patients with higher incidence rates than the rest of the country and a previously described screening experience [2,15]. Age at the time of breast cancer diagnosis was a relevant finding in our study. More than half of the patients included in our study are outside the national screening strategies [16], making their diagnosis a challenging event, thus compromising the possibility of a diagnosis at an early stage of the disease. Another important finding is the tendency to reclassify according to prognostic staging; this may be related to the immunohistochemical profile of carriers of a genetic mutation [17]. Another interesting trend relates to better survival in patients who underwent breast-conserving therapy (BCT) rather than mastectomy [18]. These findings suggest a variation in outcomes and characteristics in a group with a differential risk.

For patients with a genetic mutation, age at the time of diagnosis might be influenced by several factors, especially the diagnosis, screening approach, and genetic testing [19,20]. In our national guidance, the optimal selection of individuals for BRCA mutation testing is guided by Adelaide's criteria, which consider patient age, family breast cancer history, ovarian cancer, and a known genetic mutation [16,21]. Our observed age distribution suggests that most patients are not included in population screening methods. This age distribution is similar to other national and international reports, indicating that screening techniques may have a small contribution to detecting a mutation carrier [12-14,17,20,22,23]. Otherwise, genetic testing is linked to health information relevant to blood relatives; disseminating this information with family members (uncles, cousins, nephew/nieces) affects the probabilities for the diagnostic approach, which might explain our findings concerning consanguinity [24].

Surgical management in carriers has been controversial in the literature, specifically, the outcomes concerning the performance of BCT compared to mastectomy. Overall survival from studies with small sample sizes favors BTC over mastectomy [19]. While our findings align with this, additional studies are needed to explore the impact of other variables and surgical approach planning [17]. While BRCA1 carriers tend to have poorer survival than those with BRCA2 germline mutation, other variables influence the prognosis (e.g., clinical staging). Locally advanced breast cancer is frequently related to BRCA mutation carriers and ductal invasive tumor type but not a hormone and Her2-neu expressions [8,17]. Recently, in Colombia, Cervera et al. described the prognostic upstaging and downstaging, compared to the anatomic staging in a large Colombian breast cancer cohort. They reported that prognostic staging decreases were more frequent than those of staging increases [25]. We observed a higher frequency in the increased staging than a descent when comparing anatomical to prognostic staging. This represents the lower rates in the expression of factors that improve the prognosis in patients with breast cancer related to a genetic mutation [8,17].

According to Torres et al., “two founder BRCA1 mutations accounted for 100% of all BRCA1 mutations, and the identified founder BRCA2 mutation represented 40% of all BRCA2 mutations” [26]. In our study, most of the patients were BRCA1 and categorized as small-range BRCA1/2 founder mutations in unselected breast cancer patients. This information would improve Colombian patients' risk assessment and carrier detection [26]. This 3450del4-BIC (or c.3331_3334del-NCBI) was described as a founding mutation in Colombia in a previous study by Torres et al. [26]. In our experience, this mutation was mainly in the center municipalities of Huila, followed by 4523G>A, which was more frequent in the southern municipalities [12]. These findings may contribute to genetic screening test strategies, support efforts to understand uncertain clinically significant variants, and involve laboratories, clinicians, patients, and relatives [27]. The European origin of the mutation was introduced early in the country's colonization, resulting in a high mutation prevalence in the population, mainly in the department of Huila (Southern Colombia) [28]. In our study cohort, 105 BRCA1/2 P/LP mutation carriers were identified. The prevalence of the BRCA1 c.3331_3334delCAAG mutation was 87% (92 of 105), suggesting that specific genetic risk assessment strategies for this geographical area from Colombia need to be developed.

Our study has one of the larger sample sizes reported in the region and at the national level, including breast cancer patients associated with a genetic mutation. Our findings might represent a south Colombian region population, and it is not generalizable to other regions. A study of these patients at the national level would be of value.

## Conclusions

Women with breast cancer who carry a mutation related to breast cancer are usually younger than age 50 at diagnosis. The diagnostic approach and early detection efforts in these patients remain a challenge, given that they are not included in national strategies for breast cancer screening. This highlights the need to continue developing strategies and policies for this population. Additionally, when using the anatomical vs. prognostic system, these patients represent a risk-increased reclassification due to a higher frequency of triple-negative tumors. Surveillance for familial breast cancer does not exist in Huila. Patients with a strong family history of breast or ovarian cancers are not screened routinely, which causes preventable delays in diagnosis. The scrutiny of the prevalent BRCA1 c.3331_3334delCAAG mutation could be used as a cost-effective first approach. Patients from this region might undergo preventive measures to reduce morbidity and mortality and improve their quality of life.
